# Association of the molecular regulation of ear leaf senescence/stress response and photosynthesis/metabolism with heterosis at the reproductive stage in maize

**DOI:** 10.1038/srep29843

**Published:** 2016-07-20

**Authors:** Yi Song, Zhe Zhang, Xianjie Tan, Yufeng Jiang, Jiong Gao, Li Lin, Zhenhua Wang, Jun Ren, Xiaolei Wang, Lanqiu Qin, Weidong Cheng, Ji Qi, Benke Kuai

**Affiliations:** 1 State Key Laboratory of Genetic Engineering, School of Life Sciences, Fudan University, Shanghai 200438, China; 2 Ministry of Education Key Laboratory for Biodiversity Science and Ecological Engineering, Institute of Biodiversity Science, Fudan University, Shanghai 200438, China; 3 Maize Research Institute, Guangxi Academy of Agricultural Sciences, Nanning, Guangxi, China

## Abstract

Maize exhibits a wide range of heterotic traits, but the molecular basis of heterosis at the reproductive stage has seldom been exploited. Leaf senescence is a degenerative process which affects crop yield and quality. In this study, we observed significantly delayed ear leaf senescence in the reciprocal hybrids of B73/Mo17 and Zheng58/Chang7-2 after silking, and all the hybrids displayed larger leaf areas and higher stems with higher yields. Our time-course transcriptome analysis identified 2,826 differentially expressed genes (DEGs) between two parental lines (PP-DEGs) and 2,328 DEGs between parental lines and the hybrid (PH-DEGs) after silking. Notably, several senescence promoting genes (*ZmNYE1, ZmORE1, ZmWRKY53* and *ZmPIFs*) exhibited underdominant expression patterns in the hybrid, whereas putative photosynthesis and carbon-fixation (*ZmPEPC*)-associated, starch biosynthetic (*ZmAPS1, ZmAPL*), gibberellin biosynthetic genes (*ZmGA20OX*, *ZmGA3OX*) expressed overdominantly. We also identified 86 transcription factors from PH-DEGs, some of which were known to regulate senescence, stress and metabolic processes. Collectively, we demonstrate a molecular association of the regulations of both ear leaf senescence/stress response and photosynthesis/metabolism with heterosis at the late developmental stage. This finding not only extends our understanding to the molecular basis of maize heterosis but also provides basic information for molecular breeding.

Heterosis (hybrid vigor) refers to the phenomenon that the hybrid progeny is superior to both parents in many aspects^1^, including biomass, photosynthetic capacity, biotic/abiotic stress tolerance and grain yield^2^. Heterosis is widely exploited in the breeding of major crops, such as maize, rice, sorghum and cotton. It was estimated that the commercial grain yield was increased by about six-fold during the maize hybrid era^3^.

Heterosis has been intensively investigated in both crops and model plants. There are three classical theories aiming to explain the genetic basis of heterosis: 1) the dominance theory which assumes that the complementation of deleterious alleles contributes to heterosis^4^,^5^, 2) the overdominance theory which postulates that the favorable allelic interaction of heterozygous alleles leads to a phenotypically superior performance of F1 hybrid^6^, 3) the epistasis theory assumes that epistasis between different loci contributes to heterosis^7^. Notably, the heterotic performance seems highly variable depending on the combination of parental lines and trait(s) examined^8^. To effectively exploit heterosis in breeding, maize elite inbred lines were empirically classified into “heterotic groups” based on their capability in enhancing their hybrid’s grain yield. Analysis of the restriction fragment length polymorphism (RFLP) revealed a correlation between the hybrid’s grain yield and its parents’ genetic diversity^9^. It was reported that there were diverse polymorphism in SSR (simple sequence repeats) loci between Zheng58 and Chang7-2, which belong to different “heterotic groups”^10^. Besides, there is also a correlation between genetic diversity and their transcriptional variation^8^.

Recently, both molecular and physiological evidence suggested that photosynthesis and carbohydrate metabolism were correlated with heterosis. A physiological study in *Arabidopsis* showed that, while the rate of photosynthesis was constant per unit leaf area in both parents and hybrids, hybrids exhibited higher total photosynthesis capacity because of more chloroplasts per cell, larger cell size and leaf area^11^. Furthermore, the heterosis could be eliminated by an inhibitor of photosynthesis, and promoted by enhanced photosynthetic activity due to higher light intensity^11^. Chlorophyll (Chl) content represents a physiological marker of photosynthetic capability. Both hybrids and allopolyploids in *Arabidopsis* exhibited higher expressions of Chl biosynthesis- and starch metabolism-associated genes, which produced more Chl and starch than their parents in the same environment^12^. It was reported that differentially expressed genes (DEGs) between a super-hybrid rice and its parental lines were significantly enriched in photosynthesis and carbon fixation-associated functions^13^. Recent transcriptome studies revealed that the transcriptomic profile as well as phenotypic features in the hybrid rice was biased to those of the parent with better performance, and the epigenetic modification also contributed to the regulation of heterotic genes’ expression^14^. Other studies suggested that gene expression discrimination in the hybrid was correlated with heterotic phenotypes^13^,^15^,^16^. Genes with expression levels between those in its parents are referred as additive genes, while genes with expression levels above or below those in both of its parents are termed as non-additive genes (overdominant or underdominant, respectively)^8^. Global gene expression studies indicated that about 20% of the DEGs between parents and the hybrid exhibited non-additive modes^15^,^17^,^18^.

Maize exhibits significant heterosis in many traits, e.g. biomass, height, root growth, photosynthesis and starch metabolism, grain yield and biotic/abiotic stress resistances, and has been a model species for studying heterosis^1^,^2^. Although maize shows several heterotic traits at both vegetative and reproductive stages^2^, previous studies mainly focused on elucidating morphological and molecular phenotypes of heterosis at the early developmental stage. The heterotic phenotype and its molecular basis at the late developmental stage (post-silking and senescence stage) remain largely unexplored. Importantly, maize is sensitive to several abiotic stresses during silking and grain filling period (reproductive stage), e.g. low or high temperature and drought^19^,^20^,^21^, and post-silking stresses can hasten leaf senescence and reduce yield. Leaf senescence terminates leaf lifespan in association with massive degradation of macromolecules and redistribution of nutrients, and therefore the dynamics of the process is critical to the formation of grain yield and quality^22^. Leaf senescence is an integral part of plant development and regulated by both genetic and environment factors. Thousands of senescence-associated genes (SAGs) have been identified in *Arabidopsis*^23^, with NAC092 (ORE1) and NAC029 (NAP) being typical positive senescence regulators^24^,^25^. Several phytochrome-interacting factors (PIFs) were found to mediate both age-triggered and dark-induced leaf senescence^26^,^27^. Ethylene, abscisic acid (ABA), and jasmonic acid (JA) promote leaf senescence while cytokinin inhibits leaf senescence^28^. Previously, physiological observations showed that hybrid breeding significantly delayed maize leaf senescence during reproductive stage and extended photosynthetic period^3^; however, the molecular relationship between heterosis and leaf senescence remains elusive.

In this study, we investigated the heterotic effect on senescence regulation in maize ear leaf at the reproductive stage, using two well-known maize hybrids: B73 × Mo17 and Zheng58 × Chang7-2. All hybrids showed significantly delayed leaf senescence, larger leaf areas, higher stem heights and enhanced yields. We analyzed the dynamic changes of transcriptomes in the ear leaves of B73, Mo17 and their hybrid to explore the molecular basis of the heterosis after silking. It was found that some putative senescence regulatory genes (*ZmNYE1, ZmORE1, ZmWRKY53* and *ZmPIFs*) exhibited underdominant expression patterns in the hybrid, while those putative photosynthesis- and starch biosynthesis-associated genes (*ZmAPS1, ZmAPL*) expressed overdominantly. Several cytokinin responsive marker genes and gibberellin synthetic genes showed higher expressions in the hybrid, whereas some ethylene biosynthetic genes displayed lower expressions. Furthermore, we identified 86 differentially expressed transcription factors, including known senescence regulators, implying that they may be involved in both senescence and heterosis regulatory networks. These findings demonstrate an obvious association of delayed ear leaf senescence and enhanced photosynthesis/metabolism with reproductive heterosis in maize, which sheds light on our global understanding of the molecular basis of heterosis, and importantly provides fundamental information for developing strategies and techniques of molecular breeding in maize.

## Results

### Heterotic phenotypes of maize hybrids at the reproductive stage

**Figure 1:**
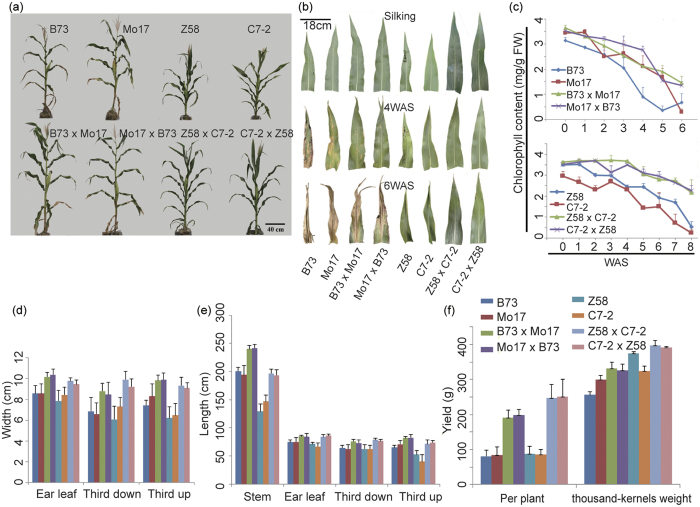
Heterotic phenotypes of maize hybrids at the reproductive stage. (**a**) The photograph shows four inbred lines and their reciprocal hybrid lines at silking stage. Z58 and C7-2 represent Zheng58 and Chang7-2, respectively. (**b**) Phenotypes of ear leaves from different lines at the indicated time points after silking (weeks after silking, WAS). (**c**) Chlorophyll contents of ear leaves from different genotypes at the indicated time points. (**d,e**) Leaf widths and lengths of different genotypes. “Third down” and “Third up” refer to the third leaves down and up from the ear leaves, respectively. (**f**) Grain yields per plant and thousand-kernel weights of different genotypes. All the analyses involve three biological replicates and error bars represent the standard deviation.

Maize B73 and Mo17 inbred lines belong to different heterotic groups and their hybrids are much taller and produce significantly higher grain yields^15^. Zheng58 and Chang7-2 are elite inbred lines developed by Chinese maize breeders in Henan Academy of Agricultural Sciences and their hybrid represents a milestone variety in China^10^. To study their heterotic performance at the reproductive stage, we generated the reciprocal hybrids of B73/Mo17 and Zheng58/Chang7-2, respectively. It was found that the reciprocal hybrids, compared with their parental lines, were significantly more vigorous, growing much taller and forming larger ear leaves (Fig. 1).

Ear leaf is the largest leaf in maize, and contributes a substantial proportion of photosynthetic products to the reproductive growth^29^. Our initial interest was therefore to investigate the phenotypic and physiological features of the ear leaves of the hybrids in comparison with those features of their parental lines at the reproductive stage (after silking). We found that the senescence process of the ear leaves was significantly delayed in the hybrids relative to their parental lines. Besides, both Zheng58 and Chang7-2 showed delayed leaf senescence relative to B73 and Mo17, and Mo17 showed an obvious delayed senescence in the ear leaves than B73. These observations were validated by measurements of the Chl content, a physiological marker of senescence as well as an index of photosynthetic capacity (Fig. 1c). Furthermore, the leaf width, leaf length and mature stem height of the hybrids all significantly exceeded those of their parents at silking stage. As expected, the reciprocal hybrids of B73 and Mo17 also had enhanced grain yields, as demonstrated by measurements of grain yields per plant and thousand-kernel weights (Fig. 1f). Similarly, the reciprocal hybrids of Zheng58 and Chang7-2 also exhibited delayed ear leaf senescence (Fig. 1a–c), as well as larger leaf areas (Fig. 1b,d,e) and higher grain yields (Fig. 1f) relative to both parents. Since the phenotypic characteristics of F1 progenies seemed not significantly influenced by the direction of the cross between male and female parents, we therefore chose the F1 hybrid of B73 (male parent) × Mo17 (female parent) and its parental lines for transcriptome profiling due to the well-studied genome of B73.

### Transcriptomic analysis of the ear leaves of B73, Mo17 and their hybrid after silking

To explore the molecular basis of the observed heterotic traits, we performed a high throughput RNA sequencing to investigate gene expression in the ear leaves of B73, Mo17 and B73 × Mo17 (shortened as BM). Total mRNA was extracted from the upper half part of ear leaves in all the genotypes, with samples of three time-points: silking (S0), one week (S1) and two weeks (S2) after silking, respectively. We obtained about 303 million high quality raw reads, 86.1% of which were mapped to the B73 reference genome APGV3, and 77.16% of mapped reads were unique mapping reads (Table 1). Gene expression levels were measured as reads per kilobase per million reads (RPKM)^30^. Each sample contained a group of genes with very low RPKM values, probably due to their low expression levels or background. We took a conservative approach and a RPKM cut-off of 0.18 as a minimal expression value to avoid false positive estimation of gene expression, producing a unimodal distribution of genes expressed in each sample (Supplementary Fig. 1). Under this criterion, we detected a total of 30,000 (75.7%) expressed genes in our samples among 39,621 reference genes, with an average of 24,999 genes (63.1% of 39,621) detected in each sample (Supplementary Table 1). We then performed pairwise comparisons to identify DEGs among the samples by GFOLD^31^, which helps to reveal the molecular mechanisms underlying the observed heterotic phenotypes.

### Transcriptomic profiling of B73 and Mo17

**Figure 2:**
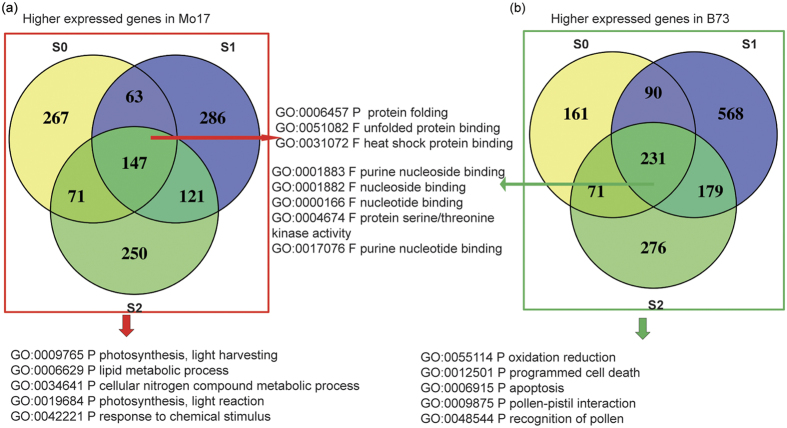
Gene ontology enrichment analysis of differentially expressed genes (DEGs) between B73 and Mo17. (**a**) 1205 genes showed higher expressions in Mo17 than B73, 147 of them were shared by all three stages. Top 5 enriched GO categories were listed in the figure, while 147 genes in (**a**) only showed enrichment of three GO categories. (**b**) 1576 genes showed higher expression in B73 than Mo17, 231 of them were shared by three stages. Top 5 enriched GO categories are listed in the figure.

It has been reported that there is a correlation between parental lines’ transcriptional variation and their genetic diversity^8^, which may constitute the genetic basis for heterosis. We identified a total of 2,746 DEGs between B73 and Mo17 (PP-DEGs) (Supplementary Table 2), with 1,205 genes showing higher expressions in Mo17 at one or multiple stages. An examination of GO annotations showed that these DEGs mainly enriched in photosynthetic process, lipid metabolic process and cellular nitrogen compound metabolic process^32^, suggesting that Mo17 may have higher photosynthetic and metabolic activities. Among the 1,205 genes, 147 were shared by all three stages discussed in our study (Fig. 2a), being enriched in three GO categories: protein folding, unfolded protein binding and heat shock protein binding. As maize plants are most sensitive to abiotic stresses at silking stage, the increased expression of these genes may imply that Mo17 is more responsive and presumably more tolerant to stresses than B73. On the other hand, 1,576 genes showed higher expression in B73 than in Mo17 at one or multiple stages (Supplementary Table 2), which were enriched in pollen-pistil interaction, pollination, programmed cell death, and apoptosis. Of those, 231 DEGs shared by three stages were enriched in nucleoside/nucleotide binding and protein serine/threonine kinase activity (Fig. 2b). This finding suggests that senescence and cell death may be initiated earlier in B73 than in Mo17 after pollination, which is consistent with the early senescence phenotype observed in B73 (Fig. 1). In summary, Mo17’s ear leaves exhibited higher photosynthetic and metabolic activities compared with B73’s after silking, which explains its relatively delayed senescence.

### Clustering of PH-DEGs and Functional Enrichment Analysis

**Figure 3:**
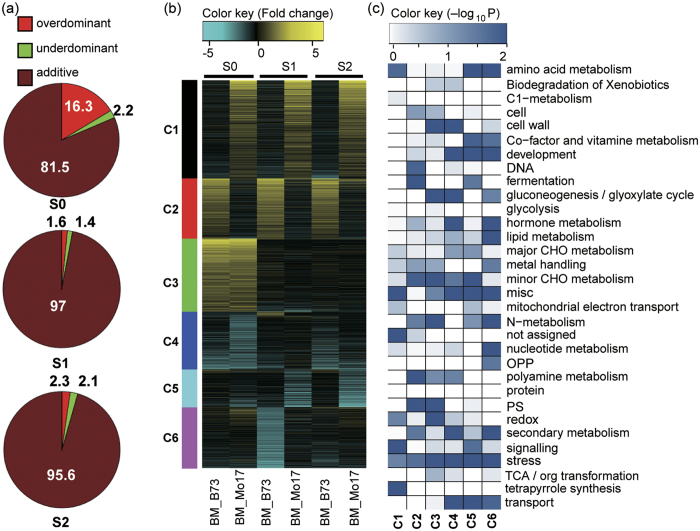
Clustering and functional category enrichment analysis of DEGs between parental lines and the hybrid (PH-DEGs). (**a**) The percentages (%) in the graph represent the ratios of additive genes and non-additive genes during S0 to S2. (**b**) Six clusters were identified along the three developmental stages using the K-Means clustering algorithm. (**c**) Functional categories enrichment (modified MapMan bins) among six clusters according to the MapMan annotations, the color key represents (−log_10_ P).

To manifest the transcriptome characteristics between the parental lines and the hybrid, we further compared the transcriptome of B73 × Mo17 hybrid to those of B73 and Mo17, respectively. We identified a total of 2,328 DEGs in the hybrid relative to at least one of its parents (PH-DEGs), representing 6.02% (2,328 out of 39,621) of the reference genes (Supplementary Table 3). Of these, 1,297, 1,198 and 924 DEGs were identified at S0, S1 or S2, respectively (Supplementary Table 4). Interestingly, among the 1,297 DEGs at S0, 211 (16.3%) were overdominant genes and 28 (2.2%) underdominant genes (Fig. 3a and Supplementary Table 4), whereas at S1 and S2, the ratio of overdominant genes dramatically decreased to ~2% (20 and 21 overdominant genes at S1 and S2, respectively, Fig. 3a and Supplementary Table 4). We further analyzed the functional enrichment of 211 overdominant genes at S0, and found that these genes were enriched in cell wall, sexual reproduction and enzyme-associated functions (Supplementary Table 5). This result, along with our previous data, demonstrated that these growth-associated and reproductive genes acted as overdominant genes at the silking stage but gradually became additive genes at S1 and S2 in the hybrid. The finding reveals that, even before phenotypic appearance of senescence initiation, the metabolism has undergone a significant decline.

To further examine the gene expression modes among different genotypes, we performed a k-means cluster analysis of the PH-DEGs (Fig. 3b and Supplementary Table 3). Among the 2,328 genes, 589 (Cluster 1, 25.3%) displayed similar expression levels between the hybrid and B73 but lower expressions in Mo17 across all stages. Numerous genes in Cluster 1 encode the proteins related to tetrapyrrole synthesis, stress, signaling, redox and amino acid metabolism according to MapMan annotation^33^. Likewise, 361 genes (15.5%) in Cluster 2 showed similar expressions in Mo17 and the hybrid, suggesting that these genes have an expression bias to Mo17 in the hybrid. These genes were enriched in growth-associated processes, such as photosynthesis, cell growth, DNA metabolism, polyamine metabolism, and N metabolism. Both C1 and C2 represent genes with similar expression levels to one parent, so PH-DEGs in C1 and C2 may also be PP-DEGs. Consistently, we found 1,528 shared genes between 2,328 PH-DEGs and 2,781 PP-DEGs (data not shown). A high similarity in the number and identity between PP-DEGs and PH-DEGs may imply that only a few or dozens of differentially regulated genes are critically responsible for the different phenotype between the two parents or between the parents and the hybrid, while majority of the genes functioning downstream are shared, possibly involved in similar pathways, in different genotypes although likely exhibiting different expression dynamics. Indeed, among the 1528 genes, 763 and 551 genes showed similar expressions in the hybrid as in B73 and Mo17 (data not shown), respectively, among the three stages, which are mainly represented by C1 and C2 according to our clustering analysis (Fig. 3). Furthermore, the clustering analysis also identified 438 genes (18.3%, Cluster 3) with higher expressions in the hybrid than in both parental lines at S1, which mainly represented the overdominant genes^17^. These genes were enriched in several growth- and metabolism-associated functions, including photosynthesis, N metabolism and amino acid metabolism, cell wall (cellulose synthesis) and redox state. We found a cell wall synthesis-associated gene *GRMZM2G089699* (homologue of *AtEXPB4*, *AT2G45110*), a pollen tube growth-associated gene *GRMZM2G125356* (homologue of *AtVGD1*, *AT2G47040*), and a putative glutathione peroxidase gene *GRMZM2G012479* (homologue of *AtGPX7*, *AT4G31870*) in Cluster 3, and all of them were overdominant genes at S0. On the other hand, Cluster 4 (346 genes, 14.8%) mainly represented the underdominant genes at S0, which were enriched in stress and secondary metabolism-associated functions. Collectively, the higher expression of growth- and metabolism-associated genes and the lower expression of stress-associated genes after silking well explain the heterotic phenotype of the hybrid.

### Enhanced expressions of Photosynthesis- and starch biosynthesis-associated genes in the hybrid

**Figure 4:**
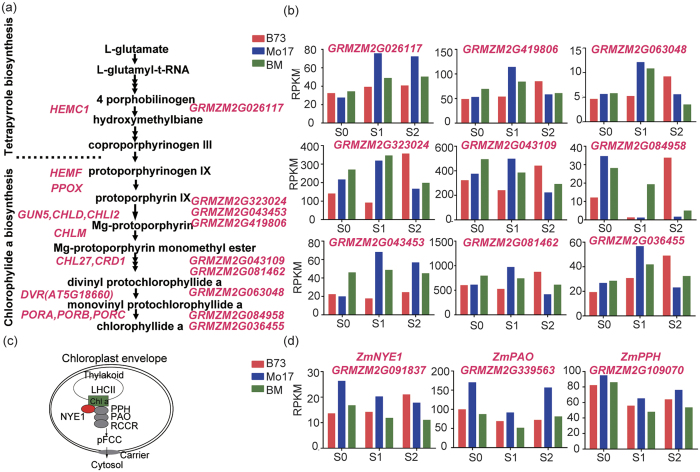
Expression levels of Chlorophyll biosynthesis- and degradation- associated genes. (**a**) Chlorophyll synthetic pathway in *Arabidopsis* and putative maize homologous genes. (**b**) Expression patterns of several putative maize chlorophyll synthetic genes. (**c**) A diagram illustrating the chlorophyll degradation pathway in *Arabidopsis*. NYE1: Non-Yellowing 1; PPH: pheophytin pheophorbide hydrolase; PAO: pheophorbide a oxygenase; RCCR: red chlorophyll catabolite reductase; pFCC: primiary fluorescent catabolites. (**d**) Expression patterns of putative *ZmNYE1*, *ZmPAO*, and *ZmPPH*.

To explore the molecular basis of the stay-green phenotype in the hybrid after silking (Fig. 1), we examined the expression of Chl biosynthesis-11 and degradation-associated genes^34^ (Fig. 4). We found that nine putative Chl biosynthetic genes, including two homologous genes of protochlorophyllide oxidoreductase B (*GRMZM2G036455*, *GRMZM2G084958*), exhibited overdominant expression patterns at one or multiple stages (Fig. 4b). Conversely, *GRMZM2G091837* (putative homologue of *AtNYE1* or *AtSGR1, At4G22920*), a critical Chl degradation regulator, and *GRMZM2G339563* (putative homologue of *AtPAO, AT3G44880*) and *GRMZM2G109070* (putative homologue of *AtPPH*, *AT5G13800*), two putative genes encoding Chl catabolic enzymes^35^, displayed underdominant expression patterns at S1 and/or S2 (Fig. 4d). Association of the elevated expression of Chl biosynthetic genes with the reduced expression of Chl catabolic genes (CCGs) well explains the stay-green phenotype of the hybrid.

**Figure 5:**
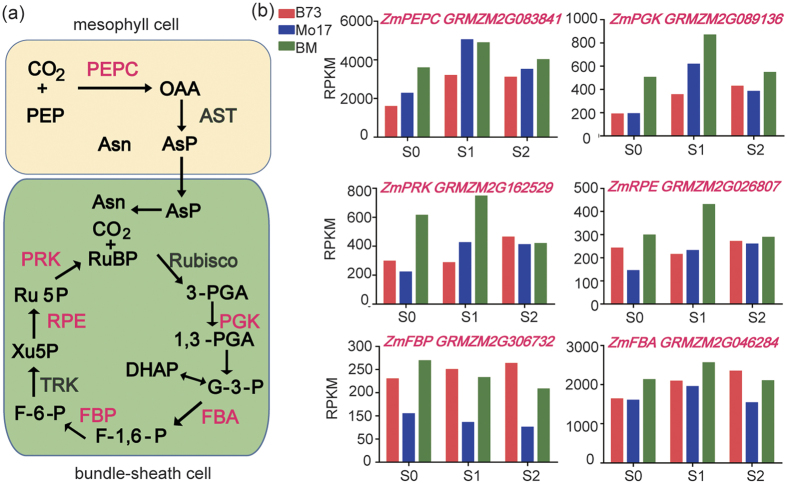
Expression levels of photosynthesis-associated enzyme genes. (**a**) A diagram shows the intermediates and key enzymes involved in calvin cycle in maize. (**b**) Overdominant expression patterns of six genes encoding carbon fixation- and calvin cycle-associated enzymes. PEP: phosphoenolpyruvate; OAA: oxaloacetate; Asp: aspartic acid; Asn: Asparagine; RuBP: ribulose-1,5-bisphosphate; PGA: phosphoglycerate; G-3-P: glyceraldehyde-3-phosphate; DHAP: Dihydroxyacetone phosphate; F-1,6-P: fructose-1,6- phosphate; F-6-P: fructose-6-phosphate; Xu5P: Xylulose 5-phosphate; AST: aspartate transaminase; Rubisco: Ribulose bisphosphate carboxylase oxygenase; PGK: 3-phosphoglycerate kinase; RPE: ribulose-5-phosphate epimerase; PEPC: phosphoenolpyruvate carboxylase; TRK: Transketolase; FBA: fructose-bisphospate aldolase; PRK: phosphoribulokinase.

Besides the higher Chl retention in the hybrid before leaf senescence, our analyses also showed that genes in the overdominant cluster (cluster 3) were enriched in photosynthesis-associated process, and many of them encoded photosynthetic proteins (Fig. 3c), including photosynthesis-associated polypeptide subunits and enzymes (Supplementary Table 6). For example, a gene encoding phosphoenolpyruvate carboxylase (PEPC) (Fig. 5), responsible for the higher efficiency of CO_2_ fixation in C4 plants^36^, showed a higher expression in the hybrid than in both parental lines at S0 and S2. Additionally, the genes coding for 3-phosphoglycerate kinase (PGK), fructose-bisphospate aldolase (FBA), fructose-1, 6-bisphosphate kinase (FBP), ribulose-5-phosphate epimerase (RPE), and phosphoribulokinase (PRK), the five enzymes required for calvin cycle (Fig. 5), also exhibited overdominant expression patterns at one or multiple stages. These transcriptomic features reveal an outline of the molecular basis for the hybrid to accumulate more dry matters during silking and grain filling period.

Starch is the main storage carbohydrate in vascular plants, and starch biosynthetic activity is conventionally considered as a measurement for photosynthetic capability. We therefore examined the expression pattern of starch synthetic genes in the hybrid. ADP-glucose pyrophosphorylase (AGP), consisting of one small subunit (APS) and one large subunit (APL), is responsible for the rate-limiting conversion of glucose-1-phosphate and ATP to inorganic pyrophosphate (PPi) and ADP-glucose^37^. The activity of AGP is allosterically activated by 3-phosphoglycerate, and inhibited by orthophosphate^38^. Recent studies revealed that overexpression of allosterically insensitive form of *AGP* homologous gene in potatoes successfully enhanced starch accumulation in tubers^39^, while silencing *AGP* gene in *Nicotiana benthamiana* reduced the starch content in leaves to 60% of those in the wild type^40^. In this study, we found that maize putative *APS* showed overdominant expression at all the stages while *APL* only showed overdominant expression at S1 (Supplementary Fig. 2). It has been reported that APL is highly unstable in the absence of APS in *Arabidopsis*^41^, and *APS1* T-DNA null mutants contain neither APL nor APS proteins^42^. It is possible that the higher expression of *APS* in the hybrid could efficiently maintain AGP complex stability and activity. *AtSnRK1* encodes the sucrose non-fermenting-1-related protein kinase, a positive regulator of starch biosynthesis, and its overexpression could lead to 30% more starch accumulation in the tubers of transgenic potato^43^. Consistently, we found that the expression level of *AtSnRK1* homologue (*AT5G21170*) in maize was significantly higher in the hybrid than in both parents at S0 (Supplementary Fig. 2), indicating that the starch biosynthetic capacity in the hybrid may be enhanced during silking and grain filling period.

### Reduced expressions of senescence-associated genes in the hybrid

**Figure 6:**
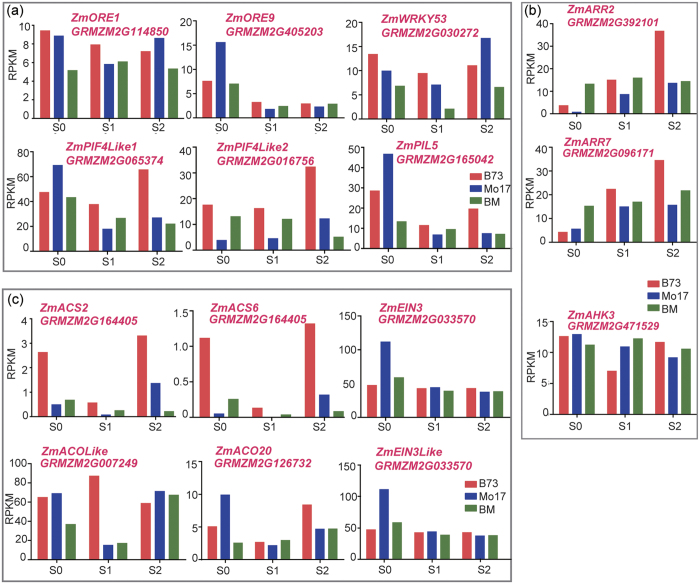
Expression levels of senescence-associated genes. (**a**) Expression levels of six putative senescence-associated positive regulators in maize. (**b**) Expression levels of putative cytokinin-related negative regulators of senescence. (**c**) Expression levels of ethylene biosynthesis- and signaling-related genes.

A recent study reported that the ear leaf initiates senescence approximately 15 days after pollination^44^. Considering that maize could finish its pollination shortly after silking, our time point S2 (two weeks after silking), may represent the time point of senescence initiation. To analyze the heterotic effect on senescence-associated genes’ expression, we compared the PH-DEGs with 4,552 previously identified senescence-induced genes in maize^44^, and identified 680 shared genes (data not shown), accounting for 29.2% of PH-DEGs. The relatively high overlap between the PH-DEGs and senescence-induced genes indicates that heterosis may affect senescence regulation or vice versa. To investigate the molecular basis of the heterosis at pre-senescence stage, we analyzed the expression of some known senescence-associated genes (SAGs) in different genotypes through S0 to S2. Two putative senescence marker genes *ZmWRKY53* (*GRMZM2G411766*) and *ZmORE1* (*GRMZM2G114850*), together with *ZmORE9* (*GRMZM2G405203*)^44^,^45^, were found to exhibit a significantly underdominant pattern in the hybrid at S0 (Fig. 6a). We further examined the expression of a few recently identified positive regulatory genes of senescence, e.g. phytochrome interacting factors (PIFs)^26^,^27^, and found that three homologous genes (*GRMZM2G065374, GRMZM2G016756*, and *GRMZM2G165042*) of *AtPIF4* (*AT2G43010*) displayed lower expressions in the hybrid than in both of the parents at one or multiple stages (Fig. 6a), indicating that the occurrence of heterosis in the hybrid is likely associated with the delay of its ear leaf senescence.

In plants, cytokinin and ethylene are two important phytohormones known to be involved in inhibiting and promoting leaf senescence^28^, respectively. As expected, three putative cytokinin response marker genes, *ZmARR2*, *ZmARR7*, and *ZmAHK3*, whose homologues known to inhibit leaf senescence in *Arabidopsis*^46^, showed overdominant expressions at one or multiple stages (Fig. 6b). Conversely, several ethylene synthetic and signaling genes exhibited lower expressions in the hybrid at one or multiple stages, including putative 1-aminocyclopropane-1-carboxylic acid synthase (ACS) and 1-aminocyclopropane-1-carboxylic acid oxidase (ACO) genes (Fig. 6c).

Although the relationship between gibberellin and senescence has not been clearly elucidated, recent studies demonstrated that gibberellins are mainly responsible for the control of leaf growth and cell division in maize. Ectopic expression of gibberellin 20-oxidase1 (GA20OX-1) could promote growth in maize^47^. In this study, we did find that two putative *ZmGA20OX* genes (*GRMZM2G368411, GRMZM2G049418*) and a putative *ZmGA3OX* gene (*GRMZM2G036340*) exhibited overdominant expression patterns at S0 (Supplementary Fig. 3), plausibly explaining the enhanced expression of growth-associated genes in the hybrid and the observed growth vigor. Collectively, the association of the higher expression of senescence inhibitory and growth promoting genes with the lower expression of senescence regulatory genes molecularly validates the delayed senescence phenotype observed in the hybrid.

### Characterization of differentially expressed transcription factors (TFs) across genotypes during silking and grain filling

**Figure 7:**
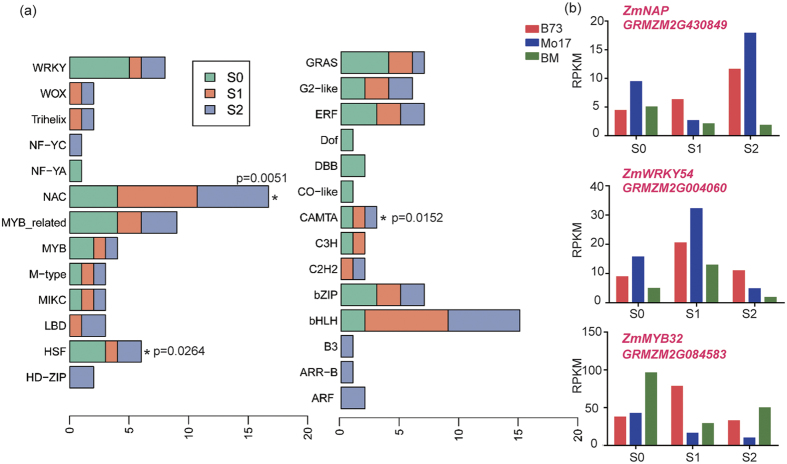
Dynamic changing inventories of differentially expressed transcription factors. (**a**) Distribution of differentially expressed transcription factor families at S0, S1 and S2. Fisher’s exact test was used to compare the ratios of TF families in our list to their ratios of total identified TF families, *p < 0.05. (**b**) Representative transcription factors with non-additive expression patterns in the hybrid.

Transcriptional regulation is one of the major regulatory modes during plant development and senescence. By referring to 3316 maize TFs recorded previously, we initially identified 1197 TFs expressed in our samples^48^. We also detected 86 TFs belonging to 26 families from PH-DEGs and they might be involved in heterosis regulation (Supplementary Table 7). By statistical significance analysis (Fisher’s exact test), we found that NAC, HSF and CAMTA families exhibited significant enrichment. NACs are widely involved in senescence regulation, and HSFs are generally regarded as stress responsive TFs. Besides, many genes of *bHLH* (11, 12.8%) were differentially expressed among the three stages (Fig. 7), presumably because they are involved in the regulation of leaf senescence and stress tolerance^26^,^49^. For example, a putative senescence regulatory bHLH gene *GRMZM2G016756* (homologue of *AtPIF4*) and a putative senescence regulatory NAC gene *GRMZM2G430849* (homologue *of AtNAP*) exhibited extraordinarily low expressions in the hybrid after silking (Fig. 7). In addition, genes of *WRKY* (8, 9.3%) and *MYB* (12, 13.9%) families were also differentially expressed (Fig. 7a). Of them was a putative *WRKY54* (*GRMZM2G004060*) that exhibited an underdominant expression (Fig. 7b). *WRKY54* was reported to show a strong but transient induction at the onset of senescence^50^. Consistently, its expression was significantly induced during S0 to S1 in all the three genotypes, but dramatically decreased at S2 (Fig. 7b). In contrast, a MYB family gene *GRMZM2G084583*, the homologue of which (*MYB32*) could respond to diverse stresses in *Arabidopsis*^51^, exhibited an overdominant expression pattern in the hybrid at S0 and S2 (Fig. 7b). We also identified four TFs with most significantly overdominant expression patterns in the hybrid: *GRMZM2G161512 (MYB), GRMZM2G083504 (bHLH), GRMZM2G381441 (ERF)* and *GRMZM2G159094 (NAC)*, and five with most significantly underdominant expression patterns: *GRMZM2G003489 (HSF), GRMZM2G159119 (MYB), GRMZM2G301485 (HSF), GRMZM2G164909 (HSF)*, and *GRMZM2G368491 (bZIP)* (Supplementary Table 7). We assumed that these TFs with significantly non-additive expression patterns are likely related to heterosis. A further GO analysis showed that 86 TFs were enriched in the regulation of multiple pathways, including cellular biosynthetic processes, nitrogen compound metabolic processes and macromolecule metabolic processes (Supplementary Table 7), suggesting that the differentially expressed TFs may regulate diverse metabolic processes. Nevertheless, solid genetic evidence is required to validate whether these TFs are directly involved in the regulation of heterosis during silking and grain filling period.

## Discussion

Maize is not only a major crop but also a model species for studying plant heterosis. Efforts have been made to elucidate the molecular basis of heterosis at its early developmental stage^8^,^15^,^17^,^18^, but the molecular analysis of its heterotic performance at reproductive stage has not been reported. In this study, we conducted a time-course transcriptome analysis of ear leaves after silking, and found that the heterosis was associated not only with the lower expression of SAGs but also with the higher expression of photosynthesis-, stress regulation- and metabolism-associated genes. This finding suggests that the mechanism of ear leaf heterosis involves both the enhancement of photosynthetic/metabolic activities, along with the stress responsiveness, and the delay of senescence. The kaleidoscopic revelation of molecular basis of the heterosis for the ear leaf helps to integrate diverse understandings of the heterotic mechanism proposed previously.

Studies in maize as well as in *Arabidopsis* and rice (*Oryza sativa*) have revealed that the yield heterosis was correlated with two critical physiological features: 1) expanded life span (delayed leaf senescence), which extends photosynthetic period^22^; 2) larger leaf area and cell size, which contain more chloroplasts and increase photosynthetic capacity^11^,^13^. Consistently, we observed a significantly delayed senescence of ear leaves in the reciprocal hybrids of both B73 vs Mo17 and Zheng58 vs Chang7-2 inbred lines, suggesting that the delay of leaf senescence may be a universal feature in maize hybrids. We also observed larger leaf areas and higher stem heights in the hybrids. Importantly, our transcriptome data revealed the lower expression of diverse SAGs (e.g. *PIFs*, *OREs*, *CCGs* and *ACSs*) and the enhanced expression of growth- (e.g. *GA20OX*s) and metabolism- (e.g. calvin cycle, chlorophyll synthesis) associated genes. The identification of heterosis-associated genes provides critical information about the molecular basis of heterotic development and maintenance at the reproductive stage.

Leaf senescence is regulated by a complex network with various regulatory nodes. Importantly, its initiation and progression facilitate massive nutrient remobilization from senescing leaves to developing organs, particularly reproductive organs^22^, the time and dynamics of which may significantly affect crop yield and quality, i.e. precocious or overly delayed initiation of leaf senescence may result in either insufficient dry matter accumulation or incomplete nutrient remobilization^52^,^53^. Maize exhibits a dual direction pattern of leaf senescence: bottom-up and top-down, with ear leaves remaining functional to the last. This characteristic leaf senescence pattern is somehow shaped presumably due to its benefiting the nutrient supply to cob development. This common feature implies that the heterosis for grain yield is likely acquired, at least in part, via optimizing the initiation and/or dynamics of leaf senescence and consequently maximizing both dry matter accumulation and nutrient remobilization during hybrid breeding. It is estimated that about 50% of total seasonal dry matter is fixed during the grain filling period in maize^54^, and it is therefore of great importance to properly modulate leaf senescence to improve the grain yield of the hybrid. In this study, we identified diverse growth vigor- and senescence-associated genes, which may be exploited for molecular breeding. *ZmNAP* (*GRMZM2G430849*), a heterosis-associated TF gene shown in our study, would have a potential application since a reduced expression of its counterpart in rice delayed leaf senescence and enhanced yield^55^. Besides, putative GA biosynthetic genes (*GA20OX*, *GA3OX*) were also identified as overdominant genes in our study, and ectopic expression of *GA20OX1* has been shown to promote growth and accumulate more vegetative biomass in transgenic maize plants, resulting in the improvement of feedstock for bioethanol production^47^. Taken together, the diverse heterosis-associated genes identified in our study may be exploitable in the molecular improvement of heterosis.

## Methods

### Plant materials

The maize B73 inbred line was originally obtained from American Germplasm Resources Information Network (www.ars-grin.gov). Mo17, Zheng58 and Chang7-2 inbred lines were kindly gifted by Dr. Huiyong Li (Cereal Crop Research Institute, Henan Academy of Agricultural Sciences, Zhengzhou, China). Plants were hand planted in 1.0-m long rows with row and plant spacing of 70 and 40 cm, respectively. The reciprocal crosspollination lines were conducted by our lab. Different genotypes were grown in the experimental field (30.940495° N, 121.127765°E) of the national center of plant gene research (Shanghai) during summer of 2011 (July 1th). The silking periods of Zheng 58/Chang7-2 hybrids were in middle August, while silking periods of B73/Mo17 hybrids and four parental lines were about 4 and 7 days later than Zheng 58/Chang7-2 hybrids, respectively. B73/Mo17 hybrids were harvested in early October and hybrids of Zheng58 and Chang7-2 were harvested in late October. Ear leaves without pathogen invasion were used for Chl content and gene expression analyses in different genotypes after silking.

### Chlorophyll content measurement

Upper half parts of ear leaves were used for Chl content quantification. Leaf samples were powdered in liquid nitrogen, and then incubated in 95% acetone/ethanol (v/v = 2:1, 5% pure water) for 12 h in darkness (−20 °C). Extracts were centrifuged at 12,000 g for 5 min at 4 °C. Chl was quantified by measuring absorbance at 645 and 663 nm, and Chl content was calculated as a ratio of (20.23*A645+ 8.023*A663) g^−1^ fresh weight. Three biological replicates were used for each measurement.

### RNA sequencing and data analysis

Upper half parts of ear leaves were ground in liquid nitrogen. We collected about 0.1 g powder in 1.5 mL tube and added 1 mL TRIzol reagent (Invitrogen) for RNA extraction according to the manufacturer’s protocol. Total RNA was treated with10U DNaseI (Ambion, USA) at 37 °C for 1 hour, and the reaction mixture was then purified with Micropoly(A) PuristTM mRNA purification kit (Ambion, USA). cDNA was synthesized from 10 μg mRNA by Superscript II reverse transcriptase (Invitrogen, USA) and sonicated into 300–500 bp fragments. The cDNA library was constructed by using TruSeq^TM^ DNA Sample Prep Kit – Set A (Illumina, USA) and amplified with TruSeq PE Cluster Kit (Illumina, USA). Sequencing was carried out on an Illumina HiSeq 2000 sequence analyzer at Chinese National Human Genomic Center at Shanghai (CHGCS). Double end 100-bp reads were generated. RNAseq data in this work have been deposited at the NCBI short read archive under accession number SRP064910.

Tophat v2.0.9^56^ was used for reads-to-reference genome alignment. Sequence reads for each sample were aligned to the maize B73 AGPv3 reference genome (http://plants.ensembl.org/Zea_mays). We chose the highest mapping score for the reads with multiple mapped positions. Transcript abundance was normalized with GFOLD v1.1.2^31^. We used 2 fold-change as cutoff (adjusted values from GFOLD), and at least one gene’s RPKM value should be higher than 1.0 for defining candidates of DEGs.

To examine different gene expression modes among different stages, k-means cluster was adopted to cluster the DEGs in different genotypes. Fold change values (adjusted values from GFOLD) between the hybrid and parental lines were used as input for clustering. Functional annotation of maize genes was carried out according to MapMan annotation^33^. Gene function enrichment analysis of DEGs was performed by using Fisher’s exact test and referring to B73 genome data. The heat map graph was constructed in R environment. In some paragraphs, we also conducted GO analyses using the AgriGO tool (http://bioinfo.cau.edu.cn/agriGO/) to facilitate comparison of overrepresented GO terms^32^.

### Orthology Analysis of Annotation for Maize genes

Orthologous and paralogous gene relationships were identified by using online website putative orthologous groups’ database (SRP064910http://pogs.uoregon.edu). We also used some orthologous or paralogous genes according to the published literatures.

## Additional Information

**Accession codes**: RNAseq data in this work were deposited at the NCBI short read archive under accession number SRP064910.

**How to cite this article**: Song, Y. *et al*. Association of the molecular regulation of ear leaf senescence/stress response and photosynthesis/metabolism with heterosis at the reproductive stage in maize. *Sci. Rep.* **6**, 29843; doi: 10.1038/srep29843 (2016).

## Supplementary Material

Supplementary Figures

Supplementary Dataset 1

Supplementary Dataset 2

Supplementary Dataset 3

Supplementary Dataset 4

Supplementary Dataset 5

Supplementary Dataset 7

## Figures and Tables

**Table 1:** Overview of mapping quality.

Genotype/stage	Total reads	Aligned reads	Unique reads	Expressed transcripts
B73_S0	34332584	30930562	23969506	25258
Mo17_S0	21066676	15709945	12662858	25093
BM_S0	40568672	35369740	26687000	25555
B73_S1	42803834	38669844	29418648	25105
Mo17_S1	37178274	31036284	23806834	24426
BM_S1	35771342	31063894	23659647	24635
B73_S2	38372646	34332615	26807905	25085
Mo17_S2	26244384	21075808	16785809	24653
BM_S2	26887434	22890669	17659278	25179
