# Supplementary material for: Association of the molecular regulation of ear leaf senescence/stress response and photosynthesis/metabolism with heterosis at the reproductive stage in maize
Source: Sci Rep. 2016 Jul 20;6:29843. doi: 10.1038/srep29843 (PMC4951735; doi:10.1038/srep29843)
Supplement: Supplementary Figures [file srep29843-s1.pdf]

Title：Association of the molecular regulation of ear leaf senescence/stress response and photosynthesis/metabolism with heterosis at the reproductive stage in maize

Yi Song[1*], Zhe Zhang[1,2*], Xianjie Tan[3], Yufeng Jiang[3], Jiong Gao[1], Li Lin[1], Zhenhua Wang[1], Jun Ren[1], Xiaolei Wang[1], Lanqiu Qin[3], Weidong Cheng[3], Ji Qi[1,2] and Benke Kuai[1,2]

Author Details

1. State Key Laboratory of Genetic Engineering, Institute of Plant Biology, School of Life Sciences, Fudan University, Shanghai, China.

2. Ministry of Education Key Laboratory for Biodiversity Science and Ecological Engineering, Institute of Biodiversity Sciences, Fudan University, Shanghai, China.

3. Maize Research Institute, Guangxi Academy of Agricultural Sciences, Nanning, Guangxi, China.

*These authors contributed equally to this work.

Correspondence and requests for materials should be addressed to B.K. (bkkuai@fudan.edu.cn) or J.Q. (qij@fudan.edu.cn)

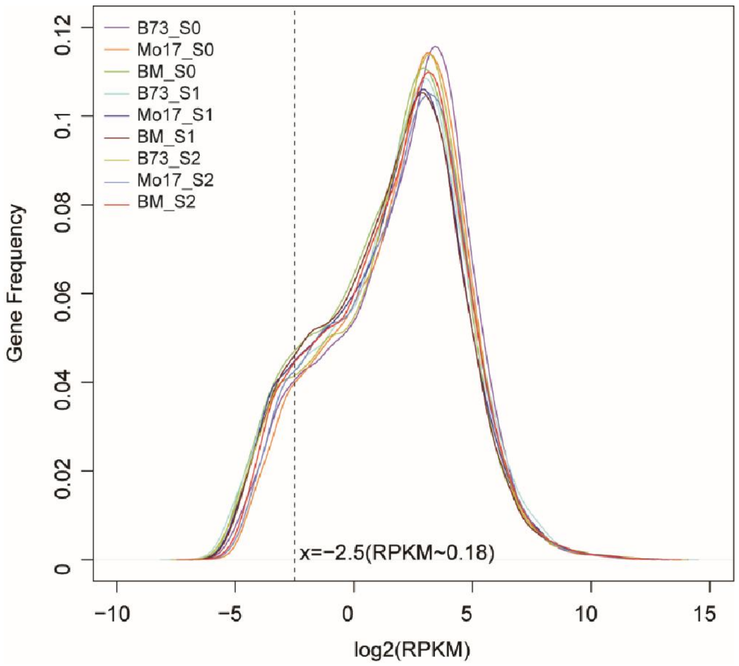

Figure S1 Gene expression values (reads per kilobase per million reads, RPKM) distribution of three genotypes at three time points.

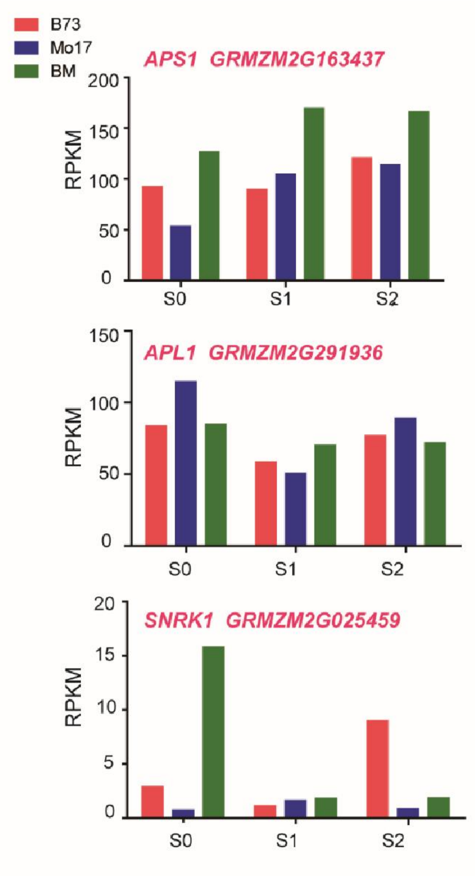

Figure S2 Expression levels of three starch biosynthesis associated genes.

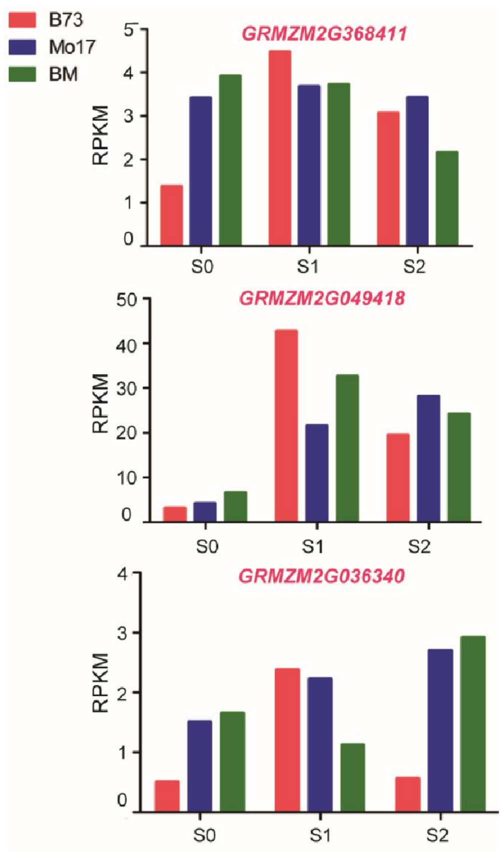

Figure S3 Expression levels of three gibberellin biosynthesis associated genes.

Table S6 Overdominantly expressed photosynthesis related genes in Cluster 3. The gene descriptions are based on mapman annotations. Values for BM/B73_S0 and BM/Mo17_S0 indicate the ratios of expression in B73 x Mo17 (BM) relative to B73 and Mo17 at S0, respectively.

GENE	BM/B73_S0	BM/Mo17_S0	Mapman annotation
GRMZM2G134130	2.0776	1.15243	PS.lightreaction.photosystem II.PSII polypeptide subunits
GRMZM2G054663	1.03267	2.24684	PS.photorespiration.glycerate kinase
GRMZM2G012397	2.0103	1.37575	PS.lightreaction.photosystem I.PSI polypeptide subunits
GRMZM2G016622	1.42642	2.11589	PS.lightreaction.photosystem I.PSI polypeptide subunits
GRMZM2G329047	2.14253	1.54632	PS.lightreaction.photosystem I.PSI polypeptide subunits
GRMZM2G016677	2.24393	1.13144	PS.lightreaction.photosystem II.PSII polypeptide subunits
GRMZM2G377855	2.94384	1.44921	PS.lightreaction.photosystem I.PSI polypeptide subunits
GRMZM2G001653	1.6317	2.26371	PS.lightreaction.photosystem I.PSI polypeptide subunits
GRMZM2G176840	1.28684	2.07918	PS.lightreaction.photosystem II.PSII polypeptide subunits
GRMZM2G451224	1.73424	2.23297	PS.lightreaction.photosystem I.PSI polypeptide subunits
GRMZM2G122337	2.67606	2.23444	PS.lightreaction.other electron carrier (ox/red).ferredoxin
GRMZM5G804323	2.18024	2.20339	PS.lightreaction.photosystem II.PSII polypeptide subunits
GRMZM2G024150	1.86143	0.666904	PS.lightreaction.photosystem I.PSI polypeptide subunits
GRMZM2G463280	0	5.481	PS.calvin cycle.PRK
GRMZM2G038365	2.07468	1.09752	PS.lightreaction.cytochrome b6/f iron sulfur subunit
GRMZM2G084279	2.19345	2.16893	PS.lightreaction.other electron carrier (ox/red).ferredoxin oxireductase
GRMZM2G067883	1.40232	2.92876	PS.lightreaction.photosystem II.PSII polypeptide subunits
GRMZM2G043162	2.43469	2.0713	PS.lightreaction.other electron carrier (ox/red).ferredoxin
